# Internight Variability of Apnea-Hypopnea Index in Obstructive Sleep Apnea Using Ambulatory Polysomnography

**DOI:** 10.3389/fphys.2019.00849

**Published:** 2019-07-09

**Authors:** Emilia Sforza, Frédéric Roche, Céline Chapelle, Vincent Pichot

**Affiliations:** ^1^EA SNA EPIS Service de Physiologie Clinique et de l’Exercice (Pole Hospitalier NOL), CHU de Saint-Étienne, Faculté de Médecine Jacques Lisfranc, Université Jean Monnet, Université de Lyon, Saint-Étienne, France; ^2^Unité de Recherche Clinique Innovation et Pharmacologie, CHU de Saint-Étienne, Hôpital Nord, Saint-Étienne, France

**Keywords:** obstructive sleep apnea – screening tools, polysomnogram (PSG), variability, hypoxic load, apnea-hypopnea index (AHI)

## Abstract

**Rationale:** Patients with obstructive sleep apnea (OSA) experience respiratory events with greater frequency and severity while in the supine sleeping position. Postural preference (associated with the sleep monitoring device) and “first night effect” could explain a night-to-night variability in OSA severity.

**Objectives:** We evaluated the variability of internight polysomnography (PSG) in a large group of OSA patients and explored factors explaining this variability.

**Methods:** 188 patients referred for probable OSA (aged 54.9 ± 11.8 y) underwent two consecutive nights of at-home PSG. The effect of age, gender, obesity, neck circumference, sleep position and sleep parameters were considered to explain changes in respiratory parameters.

**Main Results:** The mean apnea-hypopnea index (AHI) and oxygen desaturation index (ODI) were respectively, 36.3 ± 27.5 and 22.0 ± 22.7 in the first night, with a tendency to decrease during the second night. While in mild cases (5 ≤ AHI < 15) there was a significant increase in AHI related to an increase in dorsal position time during the second night, there were no changes in moderate cases (15 ≤ AHI < 30); and in severe cases (AHI ≥ 30) there was a significant decrease in both AHI and ODI during the second night independent of sleep position.

**Conclusion:** The internight variability in AHI and ODI was related to changes in sleep structure with a contribution of indices of sleep fragmentation and dorsal position. Since the changes were greater in mild OSA cases, a second night could be routinely proposed in cases with relevant clinical uncertainty.

## Highlights

- Evaluation of variability in two successive nights of at-home polysomnography. A second polysomnography is recommended to mild obstructive sleep apnea patients presenting clinical uncertainty.
- Intraindividual variability is not explained by sleep parameters and body position.

## Introduction

Obstructive sleep apnea (OSA) is now recognized as a common clinical problem, and questions of prevalence, risk and prognosis are becoming increasingly important for diagnosis and treatment. Central to these considerations is the definition of OSA, commonly defined by an Apnea-hypopnea index ≥ 5. On the basis of this cutoff, OSA is common in adults affecting 9% of women and 24% of men aged 30-60 y (Young et al., 2002a), with an even higher prevalence in the elderly (Young et al., 2002b; Neikrug and Ancoli-Israel, 2010). Moreover, untreated OSA causes sleepiness and is a major risk factor for hypertension (HT), coronary artery disease, premature mortality (McNicholas and Bonsigore, 2007; Gottlieb et al., 2010), and cognitive dysfunction (Jaussent et al., 2012). Since AHI is the primary measure used by clinicians to diagnose OSA, it is clear that a correct diagnostic procedure is fundamental to start efficacious therapy.

Some reports have evaluated the role of an internight variability in OSA patients. Chediak et al. (1996) reported that 32% of cases had a difference in AHI > 10 in two sequential nights. Le Bon et al. (2000), examining 243 subjects during sequential nights, demonstrated improved sensitivity and specificity of the results using a multiple-nights procedure. Nine patients demonstrating lack of OSA during the first night became “positive” during the second night (Dean and Chaudhary, 1992). Levendowski D.J. et al. (2009) reported a weak correlation between AHI from two polysomnographies (PSGs) conducted 40 days apart with an AHI increase of 7 events/h. In contrast, in 20 patients recorded over four consecutive nights, Bittencourt et al. (2001) did not find any significant difference in AHI through the nights with, however, a high individual variability. Overall, these studies suggest that the presence and severity of OSA may vary considerably from night-to-night, but the reasons for these are still unclear, probably related to differences in the number of patients examined, the lack of data on clinical characteristics, age, gender, sleep position, sleep structure, nocturnal hypoxemic load and the delay between nights of monitoring.

The objectives of the current study were two-fold: firstly, to examine in a large group of untreated OSA patients monitored at home for two consecutive nights to determine if there was an internight variability in OSA severity, considering the effect on both AHI and nocturnal hypoxemia; secondly, to assess which factor (i.e., clinical data, sleep position and sleep structure) mostly contributed to potential internight variability.

## Materials and Methods

### Participants

We performed a prospective analysis of patients aged 18-80 y referred to our sleep laboratory for clinical suspicion of OSA and who had two consecutive at-home ambulatory PSGs between October 2015 and October 2017. All patients accepted to participate in the study entitled “*Effectiveness of VistaO2 FLUX Device in Screening of Sleep Apnea/Hypopnea Syndrome”* (NCT02357719). Briefly, the patients had two consecutive full nights VistaO2 FLUX device monitoring synchronized with the gold standard ambulatory PSG. The present study is a substudy of the previously described VistaO2 FLUX device study.

The study was approved by the local ethics committee (CPP Sud Est 1) and all participants read and signed a written consent form. After exclusion of 30 patients with inadequate sleep data, 188 patients (51 women and 137 men) were examined.

### Clinical and Instrumental Assessment

A standardized interview was used to assess the reason to perform the sleep study, i.e., snoring, sleepiness or reported nocturnal apnea. The presence of cardiac or cerebrovascular disease, HT, diabetes, and neurological or psychiatric disorders were also collected. Subjective daytime sleepiness was evaluated using the Epworth Sleepiness Scale (ESS) questionnaire (Johns, 1991) that examines eight different situations using a 4-grade scale ranging from 0 (no chance of napping) to 3 (high chance of napping). Subjects were defined as sleepy if they had an ESS ≥ 10.

### Anthropometric Measurements

Body mass index (BMI) was calculated as kg/m^2^. Neck circumference (cm) was measured in a plane as horizontal as possible at a point just below the larynx (thyroid cartilage) and perpendicular to the long axis of the neck.

### PSG

Ambulatory PSGs (Embla^®^, Titanium) were performed at home for each patient on two consecutive nights and analyzed using Somnologica^TM^ software (ResMed, Australia). The two PSGs included four electroencephalographic (EEG) leads (C3-M2, C4-M1, O1-M2, O1-M2), of the 10-20 international electrode placement, right and left electrooculogram, chin and bilateral anterior tibialis electromyograms, electrocardiogram, sound measurement, nasal pressure, respiratory effort, body position, and oxygen saturation (SaO2) measured by pulse oximetry. For each night, patients reported the lights off and on. Sleep stages were visually scored in 30-s epochs (Kales et al., 1968). Respiratory events were manually scored according to standard criteria (Iber and American Academy of Sleep Medicine, 2007). All studies were visually validated and manually scored for respiratory events and nocturnal SaO2 according to the Chicago criteria by a single scorer (ES) with an intrascorer reliability of 89%. Hypopnea was defined as a 50% or greater reduction in airflow from the baseline value lasting ≥ 10 s and associated with at least 3% oxygen desaturation and/or EEG microarousal. Apnea was defined as the absence of airflow in the nasal cannula lasting for ≥ 10 s. The absence of rib cage movements associated with apnea defined the event as central, while a progressive increase in pulse transit time and respiratory efforts allowed definition of the episode as obstructive. AHI was defined as the ratio of the number of obstructive hypopnea and apnea events per hour of sleep. Index of total apnea, index of total hypopnea index (HI), mean apnea and hypopnea duration, and associated oxygen desaturation were also calculated. Subjects with an AHI < 5 or presenting Cheyne-Stokes breathing during at least one of the two successive nights were excluded. Indices of nocturnal hypoxemia were as follows: mean SaO2; minimum SaO2 value recorded during sleep (minimum SaO2), and oxygen desaturation index (ODI; i.e., the number of episodes of oxyhemoglobin desaturation per hour of sleep time during which blood oxygen level fell by 3% or more). Cases were subsequently stratified as mild OSA (AHI ≥ 5 but < 15), moderate (AHI ≥ 15 but < 30) and severe OSA cases (AHI ≥ 30).

### Statistical Analyses

Study population characteristics are reported as means ± standard deviation (SD) for continuous variables, and number and percentages for categorical variables. Characteristics between men and women were compared using chi-squared test for qualitative data and Student’s *t*-test for continuous data. Differences in measurements between the two nights were compared using *t*-test for paired data.

Agreement between the two nights measurements of AHI and ODI was described using Bland Altman plots. This analysis is a XY scatter plot, in which the Y-axis shows the difference between the two measurements and the X-axis represents an average of the two measurements Altman and Bland (1983). The limits of agreement were defined as the mean of the differences between the two studies ± 1.96^*^SD, with wider limits of agreement indicating greater variability. The existence of a proportional bias was determined by drawing a regression line of the two nights difference (Giavarina, 2015).

Linear correlations between ΔAHI and indices of nocturnal hypoxemia between nights and clinical parameters were searched by the least squares method. Spearman correlation coefficients (nonnormal distribution of data) were calculated and the equation of the linear regression line was given. Each correlation coefficient was associated with a scatter plot. The closer R^2^ is to 1, the stronger the linear relationship between the variables. A R^2^ equal to 0 indicates the absence of a linear relationship between the variables.

An ANCOVA was performed to test the influence of covariables on the ΔAHI and on the ΔODI between the two nights. The covariables included were defined *a priori* and was the following: age, gender, neck circumference, Δ slow wave sleep (SWS) duration between the two nights, Δ rapid eye movement (REM) sleep duration between the two nights, Δ awakening between the two nights, Δ sleep stage transition between the two nights, Δ time spent in dorsal position between the two nights, Δ time spent in lateral position between the two nights.

Data were analyzed using the SAS-Windows^®^ software version 9.4 and with MedCalc Statistical Software version 18 (MedCalc Software bvba, Ostend, Belgium; http://www.medcalc.org; 2018) for Bland Altman plots. Tests were performed at the 0.05 threshold according to a bilateral procedure. Given the multiplicity of tests for analyses according to AHI cutoff and nights 1 and 2, a 0.001 threshold test was used.

## Results

### Total Population

Table 1 shows the clinical and anthropometric data for the total sample and for men and women. Overall, the participants had an average age of 54.9 ± 11.8 y with a prevalence of men (73%) and a mean BMI of 31.4 ± 6.9 kg/m^2^. The majority of men were referred for reported snoring (38.7%) and apnea (29.2%). Almost 35% of the subjects reported HT, a cardiovascular risk factor more prevalent in men (39%). Mild OSA patients represented 21.3% of the total population, with 31.4% and 47.3% being classified, respectively, as moderate and severe cases measured during the first night. While women suffered more frequently mild (19%) or moderate OSA (35%), 43% of men were classified as severe cases. When we considered the effect of age, older subjects were found in 52% of severe OSA cases compared to 40% in middle aged patients.

**TABLE 1:** Anthropometric and clinical data for the total sample and for men and women.

	**Total**	**Men**	**Women**	***p***
n	188	137	51	
Age (y)	54.9 ± 11.8	54.3 ± 11.8	56.4 ± 11.6	0.28
BMI (kg/cm^2^)	31.4 ± 6.9	30.5 ± 5.8	33.7 ± 9.0	0.01
NC (cm)	41.2 ± 3.7	42.3 ± 3.0	38.1 ± 3.6	< 0.0001
Referred for snoring (%)	37.2%	38.7%	33.3%	0.50
Refered for apnea (%)	29.8%	29.2%	31.4%	0.77
Referred for sleepiness (%)	22.9%	22.6%	23.5%	0.90
Hypertension (%)	34.6%	32.8%	39.2%	0.41
Vascular diseases (%)	7.4%	8.8%	3.9%	0.26
Epworth Sleepiness Score	9.2 ± 5.0	9.3 ± 5.2	9.0 ± 4.6	0.98
Clinical SBP (mmHg)	137.4 ± 16.7	140.1 ± 16.1	135.6 ± 17.0	0.001
Clinical DBP (mmHg)	85.1 ± 8.3	86.9 ± 8.6	83.9 ± 8.0	0.002

*BMI, body mass index; NC, neck circumference; SBP, systolic blood pressure; DBP, diastolic blood pressure. P, independent *t*-test or chi-square between men and women.*

Table 2 shows the sleep and respiratory data for the first and the second nights in the entire sample. A “first night effect” was found with a significant increase in slow wave and REM sleep, and a decrease in light sleep and sleep stage transition that is considered as indices of sleep fragmentation. Mean AHI and ODI were not significantly altered between nights.

**TABLE 2:** Sleep and respiratory data during the first and the second nights for the total sample.

	**First night**	**Second night**	***p***
Total sleep time (min)	403.1 ± 83.2	411.5 ± 78.2	0.20
WASO (min)	116.4 ± 96.1	114.0 ± 97.1	0.61
Sleep efficiency (%)	76.7 ± 13.1	78.1 ± 11.6	0.14
Awakenings (nb)	93.9 ± 55.4	90.2 ± 52.1	0.33
Sleep stage transition (nb)	318.5 ± 159.9	294.4 ± 158.5	0.03
Sleep latency (min)	12.8 ± 19.1	10.7 ± 16.0	0.22
Light sleep (min)	300.1 ± 70.3	283.4 ± 66.9	0.004
SWS sleep (min)	44.6 ± 33.4	59.2. ± 44.3	< 0.0001
REM sleep (min)	57.5 ± 32.3	67.4 ± 31.4	< 0.0001
Apnea index n/h)	18.2 ± 23.7	15.9 ± 18.5	0.14
Hypopnea index (n/h)	17.9 ± 14.7	17.5 ± 12.2	0.60
Apnea+hypopnea index (n/h)	36.2 ± 27.6	33.4 ± 22.1	0.08
Apnea duration (s)	17.2 ± 4.6	17.7 ± 5.2	0.10
Hypopnea duration (s)	14.8 ± 2.4	15.3 ± 2.7	0.004
Apnea desaturation (%)	87.7 ± 4.3	88.0 ± 4.0	0.29
Hxpopnea desaturation (%)	89.5 ± 3.9	89.9 ± 3.4	0.02
ODI (nb/h)	22.0 ± 22.7	18.1 ± 15.8	0.002
SaO_2_ min (%)	78.5 ± 8.5	79.6 ± 8.0	0.01
SaO_2_ mean (%)	92.0 ± 2.9	92.2 ± 2.5	0.09

*WASO, wake after sleep onset; Light sleep, stages 1 and 2; SWS, slow wave sleep; REM, rapid eye movements sleep; ODI, oxygen desaturations index; SaO_2_, oxygen saturation. P, paired *t*-test between the first and the second nights.*

### Sleep and Respiratory Data During the Two Consecutive Nights According to OSA Severity

**FIGURE 1:**
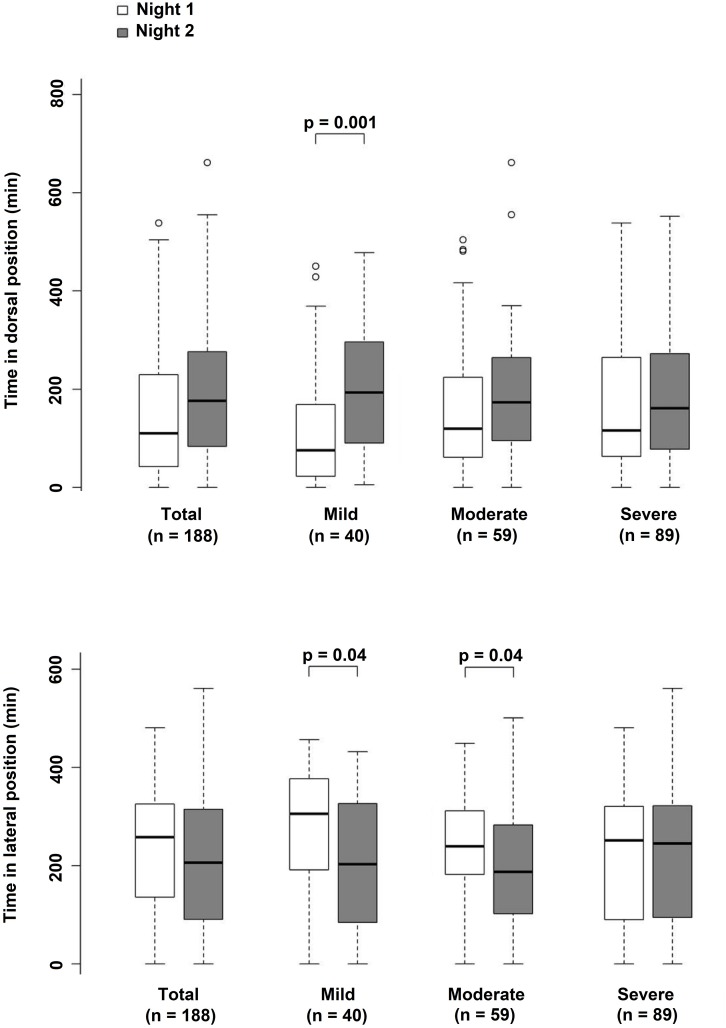
Histograms showing the time spent in the dorsal and ventro-lateral positions in the overall group and in the three groups stratified according to apnea-hypopnea index (AHI).

To assess if the severity of OSA in the first night affected internight respiratory changes, we reported sleep and respiratory data among the three groups of cases (Table 3). Significant sleep and respiratory alterations appeared between night 1 and night 2 according to OSA severity. In mild cases, there were no significant differences between nights for sleep parameters. In contrast, a significant increase in AHI (from 9.4 ± 3.0 to 16.7 ± 11.6 n/h, *p* = 0.001) was found to be associated with an increase in time spent in the dorsal position (Figure 1, *p* < 0.0001). In severe cases, there was a strong and significant increase in SWS (from 35.3 ± 35.5 to 58.6 ± 51.8 min, *p* < 0.0001) and REM sleep durations (from 46.2 ± 31.1 to 63.6 ± 29.0 min, *p* < 0.0001) without differences in sleep position (Figure 1) but were associated with a significant decrease in AHI (from 57.9 ± 25.7 to 45.4 ± 23.1 n/h, *p* < 0.0001) and ODI (from 36.7 ± 24.6 to 25.8 ± 17.9 n/h, *p* < 0.0001). There were no significant changes in sleep parameters and position in moderate cases.

**TABLE 3:** Polysomnographic data for the three groups of patients stratified according to the apnea-hypopnea index and the two nights.

	**5 ≤ AHI < 15 *n* = 40**			**15 ≤ AHI < 30 *n* = 59**			**AHI** ≥ **30 *n* = 89**		
	**1st night**	**2nd night**	***p***	**1st** **night**	**2nd night**	***p***	**1st night**	**2nd night**	***p***
Total sleep time (min)	404.0 ± 61.1	413.4 ± 70.4	0.43	407.4 ± 87.3	402.8 ± 75.6	0.67	399.8 ± 89.3	416.4 ± 83.4	0.11
WASO (min)	102.8 ± 85.0	97.3 ± 99.5	0.59	106.1 ± 104.0	108.9 ± 98.9	0.76	129.3 ± 95.0	124.8 ± 94.5	0.50
Sleep efficiency (%)	78.5 ± 10.8	79.9 ± 11.7	0.47	79.0 ± 13.3	78.4 ± 11.4	0.73	74.3 ± 13.5	77.0 ± 11.7	0.05
Awakenings (n)	81.1 ± 34.8	82.3 ± 46.5	0.87	75.5 ± 36.1	76.6 ± 38.6	0.80	112.0 ± 67.2	103.0 ± 59.3	0.19
Sleep stage transition (n)	254.8 ± 83.5	247.4 ± 110.6	0.62	248.9 ± 93.8	246.0 ± 108.0	0.79	394.1 ± 186.3	348.3 ± 186.9	0.04
Sleep latency (min)	13.1 ± 19.4	10.9 ± 18.0	0.56	14.1 ± 24.5	12.7 ± 18.1	0.72	11.9 ± 14.4	9.3 ± 13.5	0.15
Light sleep (min)	288.4 ± 57.4	281.0 ± 55.7	0.41	280.5 ± 70.4	267.7 ± 61.3	0.12	318.3 ± 71.5	295.0 ± 73.1	0.02
SWS (min)	49.4 ± 27.8	58.5 ± 35.4	0.07	55.3 ± 30.0	60.5 ± 37.6	0.15	35.3 ± 35.5	58.6 ± 51.8	< 0.0001
REM sleep (min)	64.3 ± 28.7	72.2 ± 32.7	0.12	69.9 ± 31.0	69.8 ± 33.7	0.99	46.2 ± 31.1	63.6 ± 29.0	< 0.0001
Apnea index (n/h)	1.9 ± 2.0	5.1 ± 7.1	0.01	6.7 ± 4.9	10.6 ± 11.4	0.01	33.6 ± 27.2	24.2 ± 21.8	0.004
Hypopnea index (n/h)	7.5 ± 3.0	11.4 ± 6.1	0.002	15.3 ± 5.3	15.9 ± 8.3	0.53	24.3 ± 18.5	21.2 ± 14.8	0,05
Apnea+hypopnea index (n/h)	9.4 ± 3.4	16.7 ± 11.6	0.001	22.0 ± 4.2	26.5 ± 14.9	0.02	57.9 ± 25.7	45.4 ± 23.1	< 0.0001
Apnea duration (sec)	15.2 ± 3.3	15.3 ± 3.2	0.85	15.7 ± 3.4	16.6 ± 4.9	0.10	19.2 ± 5.0	19.5 ± 5.5	0.44
Hypopnea duration (sec)	14.2 ± 2.5	14.1 ± 1.8	0.91	14.7 ± 1.9	15.1 ± 1.9	0.09	15.3 ± 2.6	16.1 ± 3.3	0.01
Apnea desaturation (%)	88.3 ± 2.4	88.7 ± 2.2	0.25	89.3 ± 3.6	88.9 ± 4.3	0.40	86.4 ± 4.9	87.1 ± 4.2	0.10
Hypopnea desaturation (%)	90.6 ± 2.6	90.6 ± 2.0	0.98	90.7 ± 2.7	90.8 ± 2.3	0.83	88.2 ± 4.6	89.0 ± 4.3	0.01
ODI (%)	4.6 ± 4.2	9.4 ± 9.9	0.01	11.6 ± 9.2	12.5 ± 8.6	0.54	36.7 ± 24.6	25.8 ± 17.9	< 0.0001
SaO_2_ min (%)	82.6 ± 5.5	82.6 ± 4.2	0.95	81.5 ± 6.4	82.2 ± 5.6	0.30	74.7 ± 9.1	76.6 ± 9.4	0.02
SaO_2_ mean (%)	92.6 ± 2.3	92.7 ± 2.0	0.51	93.1 ± 1.5	92.8 ± 2.0	0.21	91.0 ± 3.4	91.6 ± 2.9	0.01

*WASO, wake after sleep onset; Light sleep, stages 1 and 2; SWS, slow wave sleep; REM, rapid eye movements sleep; ODI, oxygen desaturations index; SaO_2_, oxygen saturation. P, paired *t*-test between the first and the second nights.*

According to these changes, the percentage of patients classified as mild cases fell from 28% to 21% from night 1 to night 2, moderate cases increased from 32% to 34%, and severe OSA cases decreased from 46% to 43%; overall indicating greater changes for mild OSA cases.

### Bland Altman Plots

**FIGURE 2:**
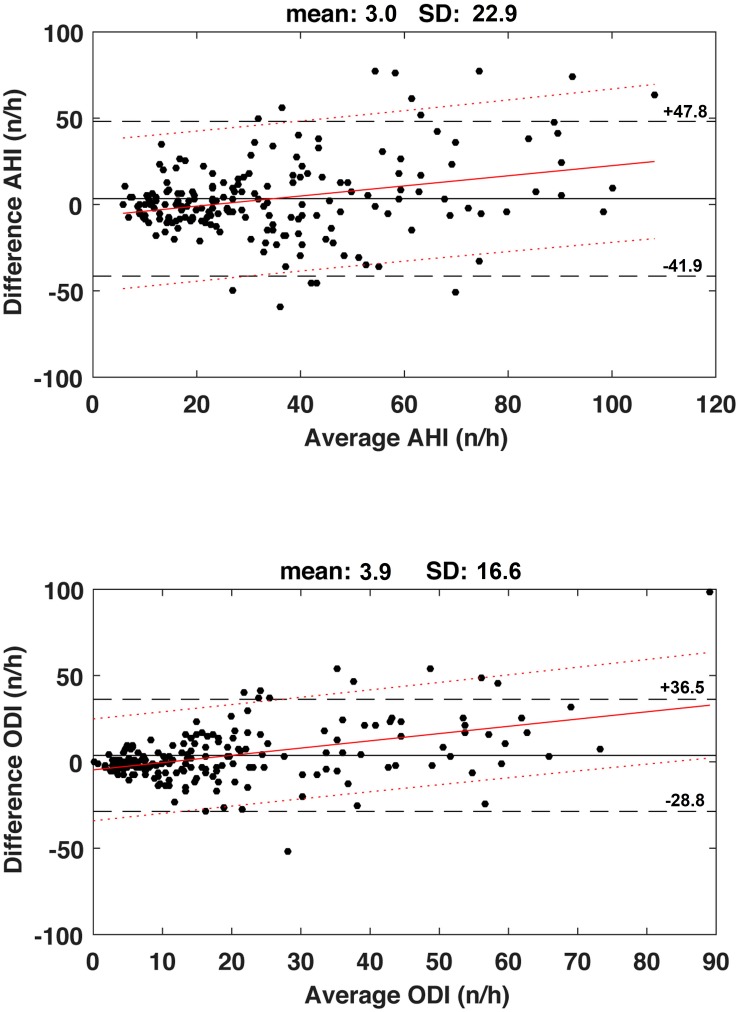
**Upper panel.** Bland Altman plots for the apnea-hypopnea index (AHI) values measured during the first and second nights. Horizontal solid line is the mean value and dashed lines represent the limits of agreement (mean ± 1.96SD). Continuous red line represents the regression line and dotted red lines are the 95% confidence interval limits. Regression lines: *Y* = 0.293^*^X-6.785, *r*^2^ = 0.082, *p* < 0.001. **Lower panel.** Bland Altman plots for the oxygen desaturation index (ODI) values measured during the first and second nights. Horizontal solid line is the mean value and dashed lines represent the limits of agreement (mean ± 1.96SD). Continuous red line represents the regression line and dotted red lines are the 95% confidence interval limits. Regression line: *Y* = 0.420^*^X-4.600, *r*^2^ = 0.198, *p* < 0.001.

The Bland Altman plots (Figure 2) show a mean difference between day 1 and day 2 of +3.0 ± 22.9 n/h with 95% limits of agreement ranging from −41.9 to +47.8 n/h for AHI, and +3.9 ± 16.6 n/h with 95% limits of agreement ranging from −28.8 to +36.5 n/h for ODI. For both indices, there were positive linear regression between differences and severity with very poor coefficients of determination (AHI: *r*^2^ = 0.082, ODI: *r*^2^ = 0.198, both *p* < 0.001).

### Regression and ANCOVA Analyses

Spearman’s correlation analysis revealed that ΔAHI was significantly related to ΔODI (*r*^2^= 0.557, *p* < 0.0001), ΔSWS duration (*r*^2^= 0.123, *p* < 0.0001), ΔREM sleep duration (*r*^2^= 0.095, *p* < 0.0001), Δ in awakening (*r*^2^ = 0.133, *p* < 0.0001), Δ in sleep stage transition (*r*^2^= 0.187, *p* < 0.0001), and Δ in the time spent in dorsal (*r*^2^= 0.129, *p* < 0.0001) and lateral (*r*^2^= 0.079, *p* < 0.0001) positions. No significant relationship was found between ΔAHI and age, gender, neck circumference, and ESS score.

Regarding the variation of AHI between nights 1 and 2 (Table 4), ANCOVA analysis revealed that the most important factors were the AHI of night 1 (*p* < 0.0001) and the time spent in dorsal position on night 1 (*p* = 0.0004). Regarding the variation in ODI between nights 1 and 2, ANCOVA analysis revealed that the most impostant factors were the ODI of night 1 (*p* < 0.0001) and the time spent in dorsal and lateral positions on night 1 (*p* < 0.0001 and *p* = 0.0006, respectively).

**TABLE 4:** ANCOVA regarding the variation between first and second night of AHI and ODI.

**Covariables**	**Estimated parameter**	**Standard error**	***p*-value**	**Estimated parameter**	**Standard error**	***p*-value**
Age	0.125	0.125	0.32	–0.018	0.080	0.82
Gender	–3.235	3.768	0.39	–1.284	2.429	0.60
Neck circumference (cm)	–0.182	0.475	0.70	0.031	0.306	0.92
AHI night 1	–0.482	0.068	< 0.0001	–0.518	0.052	< 0.0001
SWS duration night 1	0.102	0.051	0.045	0.078	0.032	0.01
REM duration night 1	0.007	0.057	0.90	0.028	0.036	0.43
Awakening night 1	–0.001	0.043	0.98	–0.017	0.027	0.54
Sleep stage transition night 1	0.006	0.015	0.70	0.016	0.009	0.09
Time spent in dorsal position 1	–0.052	0.014	0.0004	–0.041	0.009	< 0.0001
Time spent in lateral position 1	–0.037	0.014	0.01	–0.033	0.009	0.0006

*AHI, Apnea hypopnea index; SWS, slow wave sleep; REM, rapid eye movements sleep. The same results are observed if the Body Mass Index is included instead of neck circumference.*

## Discussion

The present study was conducted to evaluate the internight variability in respiratory and sleep parameters in a large group of OSA patients examined on two consecutive nights and to point out the factors involved in the potential variability.

The first finding of the current study was the lack of a major difference in AHI and ODI in the overall group, with a tendency to observe a lower AHI and less severe hypoxemic load during the second night of PSG monitoring. The second finding was that greater and significant variability was present in mild cases, which was explained by an increase in time spent in the dorsal position. While moderate cases did not differ for sleep and respiratory data, in severe cases there was a significant decrease in AHI and ODI, which were unrelated to sleep position but correlated with an improvement in sleep structure. Thus, we can say that the reported variability in OSA patients greatly affected mild cases, with sleep position and improvement in sleep structure playing a key role on the night-to-night variability in OSA severity.

The results of our study are partially in contrast to previous reports related to differences in the size sample, the analysis of changes not only in AHI but also in ODI and sleep structure, and the analysis on sleep position, neglected in previous reports. Ahmadi et al. (2009) considered 193 patients who spent two nights in hospital, but the interval between studies was not defined. As observed in our group of patients, the mean AHI in their study did not differ between nights (6.6 vs. 6.9 n/h); but there was a greater standard deviation during the second night, interpreted as reflecting a higher variability. However, their patients were mild OSA cases; moderate or severe cases were not examined in their study. Bliwise et al. (1991) examined 71 elderly volunteers with OSA on two consecutive in-laboratory nights. They found 13 subjects with high variability and 58 with low variability, suggesting that in the elderly a low internight variability was present. These results were in line with those reported by Wittig et al. (1984) and Mendelson (1994), which did not find any internight differences in OSA severity. It seems that the controversy between results could be explained by the lack of data on sleep position in earlier studies and analysis was restricted to mild cases.

The internight changes in body position and sleep patterns result from an individual’s maladaptation to the unfamiliar PSG device and sensors. Also, the between and within night variations in the minimum amount of positive pressure required to maintain an unobstructed upper airway during inspiration are concordant with the internight changes (Bliwise et al., 1991). In addition to the “first night effect” and physiological inconstancy, the scoring changes can also be explained by differences in scoring or measurement error. Indeed, both effects of internight differences in signal quality and the well known intra- and interscorer discrepencies (Whitney et al., 1998) will amplify the variability. Interestingly enough, the relatively poor correlations between respiratory events and night parameters to explain the differences between nights may support the fact that the “first night effect” is reduced for home PSG compared to in-hospital tests.

Although speculative and based on our results and previous studies, we suggest that a high variability should be suspected in mild patients with a typical clinical OSA profile in whom a lower AHI during the first night is related to reduced time spent in the dorsal position during this night. If so, a second PSG should be recommended. A second night should be proposed also for severe OSA cases with a very bad sleep observed in the first PSG, in that an overestimation of the AHI severity might occur, affecting the estimation of therapy outcomes.

Finally, our results stress the need to perform comparative analysis of the indices of hypoxemia that allow a better estimation of OSA variability and severity.

This study included a relatively large sample size with a representative number of men and women, and the analysis was conducted through two consecutive nights performed at-home to reduce the “first night effect” and disturbed sleep in hospital (Levendowski D. et al., 2009). We cannot exclude a regression to the mean effect for the subgroups analyses since these severity groups were determined by the first night’s AHI measurement. However, this statistical phenomenom is minor since some statistical analyses showed significant variations between nights 1 and 2 when considering the whole data set. Also, one can add that the two measurements were not carried out under the same conditions since the subjects became accustomed to wearing the polysomnographic recorder on the second night. We did not present the results concerning central apneas since the goal of the study was to focus on an obstructive apnea population. Globally, the remaining subjects who presented several central apneas reached indexes around 1 to 3 central apneas per hour. Moreover, these indexes were relatively stable over the two successive nights and thus did not influence significantly the number and variability of the AHI. Also, the lack of a control group as patients with an AHI < 5 must be mentioned as limitations.

## Conclusion

A night-to-night variability in OSA severity may be a confounding factor in assessing diagnosis, assessment of disease severity, and treatment outcomes. The difference in the two nights in mild and severe cases may possibly explain why some patients do not experience improvement in daytime function and adherence to continuous positive airway pressure treatment.

This study has demonstrated that in a large clinical OSA population there were no strong significant differences in AHI and indices of hypoxemia during two consecutive nights for moderate to severe OSA cases; the most significant changes were found in mild cases for which repeated measurements are needed to estimate the real severity of the disease and improvement in therapy. Finally, since internight OSA variability is mostly explained by the effect of sleep structure and sleep position, clinicians should consider these variables in the interpretation of PSGs to make the best diagnosis and adequate treatment.

## Author’s Note

ES sadly passed away abruptly in October 2017 at the end of writing this manuscript.

## Ethics Statement

This study was carried out in accordance with the recommendations of name of guidelines, name of committee with written informed consent from all subjects. All subjects gave written informed consent in accordance with the Declaration of Helsinki. The protocol was approved by Comite de Protection des Personnes Sud Est 1 (Saint-Étienne, France).

## Author Contributions

ES conceived the study, performed the hypothesis delineation, analyzed the data, and prepared the manuscript. FR conceived the study, acquired and analyzed the data, and prepared the manuscript. CC performed statistical analyses and revised the manuscript. VP carried out the statistical analysis of the data and prepared the manuscript.

## Conflict of Interest Statement

The authors declare that the research was conducted in the absence of any commercial or financial relationships that could be construed as a potential conflict of interest.
